# The importance of length and area estimate for forensic medicine without using a measurement tool

**DOI:** 10.55730/1300-0144.5709

**Published:** 2023-08-11

**Authors:** Ahmet TURLA, Hüseyin KARA, Nilay CANKURT AYAR, Berna AYDIN, Fulya Zeynep LEVENT, Oğuzhan KÜLBAZ

**Affiliations:** 1 Department of Forensic Medicine, Faculty of Medicine, Ondokuz Mayıs University, Samsun, Turkiye; 2 Sancaktepe Şehit Prof. Dr. İlhan Varank Training and Research Hospital, İstanbul, Turkiye

**Keywords:** Measuring, estimating, forensic report, forensic error

## Abstract

**Background/aim:**

Accurately measuring and recording the length or area of lesions affects the judgement of the forensic report, surgical wound management and, in some countries, the billing of health care services. The aim of this study was to determine whether the length and area of lesions described by physicians by estimation are accurate.

**Materials and methods:**

This study was designed as a cross-sectional descriptive study and was conducted with 494 participants consisting of internists and physicians at Ondokuz Mayıs University, Faculty of Medicine. The participants were asked to estimate the lengths or areas of 6 different shapes in the questionnaire form without using a measuring instrument.

**Results:**

Of the participants, 216 (43.7%) were interned physicians and 278 (56.3%) were physicians. Most 122 people (24.7%) answered the curved line shape with a length of 4 cm as “exact value”. The average of the values given by the internists to each shape was higher than the average of the values given by the physicians to each shape and was found to be further away from the true value. It was determined that more than half of the participants gave values above the actual length and area values of the shapes. It was observed that the rate of correct estimation of line shapes was higher than the rate of correct estimation of area shapes both as exact values and with ±10% margin of error. For line shapes, it was observed that the number of those who gave exact values decreased as the line length increased.

**Conclusion:**

When preparing forensic reports, determining surgical wound management and billing, estimated data should not be used in lesion description. It would also be useful to develop tools that will enable physicians to make measurements in terms of easy use.

## 1. Introduction

Estimation is defined as developing a quick and acceptable idea about the quantity or size of something without actually counting or measuring it [1]. We frequently use length and area estimations in many areas throughout our lives. However, studies have shown that people have difficulty in estimating measurement and their estimates are often far from the actual measurement [2].

Situations that occur as a result of external factors, which impair the mental or physical health of individuals or cause their death are characterised as forensic cases [3]. One of the main duties of physicians is to prepare a forensic report after providing the necessary medical assistance and notification in forensic cases [4]. In the forensic reports, traumatic lesions are described in detail, the characteristics of the instrument causing the lesion and the features necessary to determine the age of the wound are defined [5]. The length of the lesions, the ratio of lesions and burns to the body surface area, the diameter of the hair loss area due to trauma, the area of flap-style injury on the scalp affect the conditions of the injury in our legal regulations such as “whether or not it causes a life-threatening situation”, “whether or not it is a mild injury that can be eliminated with a simple medical intervention” etc. [6,7]. Errors made during the preparation of the forensic report may mislead the judicial authorities and lead to a miscarriage of justice [8]. In one study, it was reported that detailed description of lesions was not made in 432 (57.6%) of 750 cases in which a forensic report was prepared and in 169 (69.3%) of 244 cases in another study [8, 9]. In forensic examinations, it is obvious that reports prepared on the basis of estimations without due care will be erroneous [8].

In addition, lesion length affects surgical wound management or billing in some countries such as the United States of America. Lesion lengths determined by estimation may lead to incorrect billing and surgical applications [2, 10–12].

The aim of this study was to determine whether the length and area of the lesions defined by physicians by estimation without using a measuring instrument while defining the lesions found in the patients they examined and preparing forensic reports were correct or not.

## 2. Materials and methods

This cross-sectional descriptive study was carried out between 01.05.2022–31.05.2022 with a total of 494 people, including 216 intern physicians at Ondokuz Mayıs University Faculty of Medicine and 278 assistant and specialist physicians working in the same university hospital. The prepared questionnaire form was applied to 10 physicians working at Ondokuz Mayıs University Faculty of Medicine and its functionality was evaluated and finalised in line with the suggestions. The questionnaire form consisting of nine questions was applied face-to-face to all interns and physicians who could be reached between the specified dates after their verbal consent. In the questionnaire form; physicians were asked to answer some descriptive questions such as age, gender, intern or physician status, duration of practice, and six questions in total to evaluate length, diameter and area estimation without using a measurement tool. No intervention was made for physicians who measured length, diameter and area with a finger or by proportioning.

Participants who were working as interns or physicians at Ondokuz Mayıs University Faculty of Medicine, who agreed to participate in the survey and answered all the questions were included in the study. No participant was excluded from the study.

Taking into account the study by Peterson et al. [10], the relationship between the length estimated by the participants and the actual length was evaluated using the G*Power 3.1.9.4 programme, and it was determined that a total of 45 cases should be included in the study with 95% confidence (1-alpha) and 95% test power (1-ß).

The data obtained were analysed using IBM SPSS Statistics V26.0 software (IBM Corp. in Armonk, NY.). Whether the numerical data showed normal distribution was analysed by Kolmogorov Smirnov test, QQ plot, skewness and kurtosis statistics. It was determined that the numerical data did not show normal distribution. Frequency analyses, chi-square test for comparisons between groups, Mann-Whitney U test for comparing numerical data with categorical data, Spearman Correlation analysis for correlation analysis were used statistically. The significance level was accepted as p < 0.05. The study was performed according to the Helsinki Declaration Ethical principles.

## 3. Results

The same data or information given in a Table must not be repeated in a Figure and vice versa. It is not acceptable to repeat extensively the numbers from Tables in the text or to give lengthy explanations of Tables or Figures.

The mean age of 494 participants was 27.55 ± 5.47 years (min: 21–max: 67), 245 (49.6%) were male and 249 (50.4%) were female. Of the physicians, 216 (43.7%) were intern physicians and 278 (56.3%) were physicians, and the mean duration of their professional career was 5.32 ± 5.96 years.

**Figure 1:**
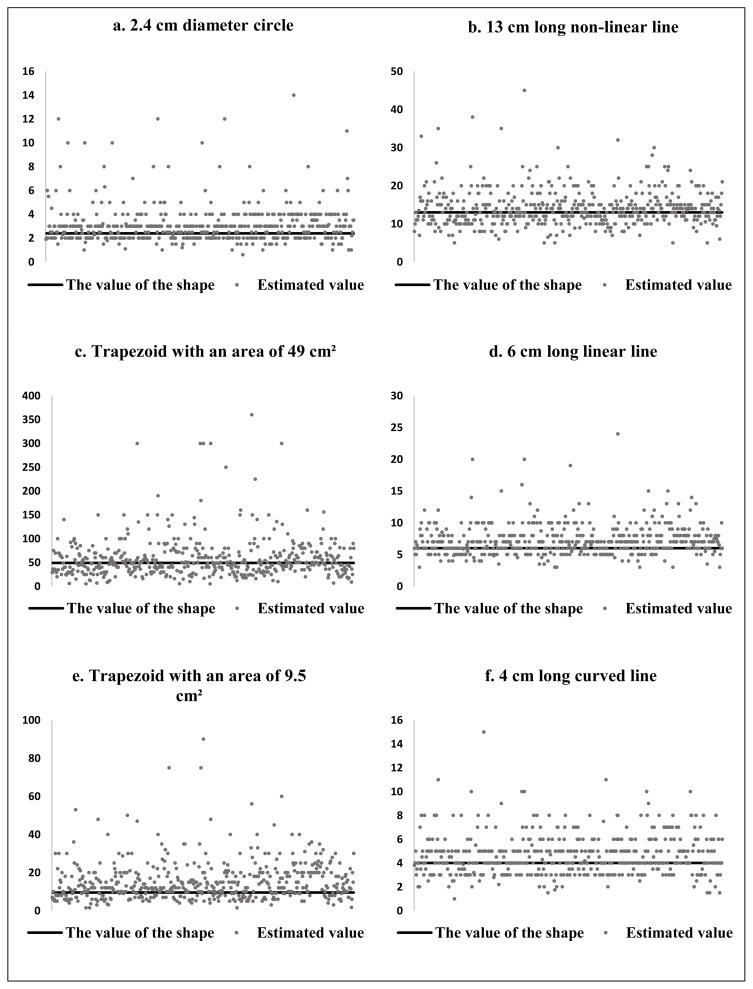
(a–f). Distribution of the values given by all participants to lines and shapes.

The comparison of the mean values given by interns and physicians in the questionnaire form is shown in Table 1. A statistically significant difference was found between the mean values given by interns and physicians for the trapezoid with an area of 9.5 cm^2^ (U = 25.419.5, p = .003), circle with a diameter of 2.4 cm (U = 26.620, p = .027) and curved line with a length of 4 cm (U = 26.776, p = .036), but no statistically significant difference was found between the values given for the other shapes. In these three figures, the mean values given by interns were higher than those given by physicians and showed more deviation from the actual value. Although not statistically significant, the mean of the values given by the intern physicians was higher and deviated more from the actual value than the physicians in the other figures. There was no statistically significant difference between gender and the mean values given for the figures. The distribution of the values given by the participants for each figure is shown in Figure (1a–1f).

Among those who filled out the questionnaire, maximum 122 people (24.7%) answered the curved line shape with a length of 4 cm and minimum 1 person (0.2%) answered the trapezoid shape with an area of 9.5 cm^2^ as “exact value”. The error rates of the values given by all participants (n: 494) are given in Table 2. No one answered correctly for all length and area measurements.

**Figure 2:**
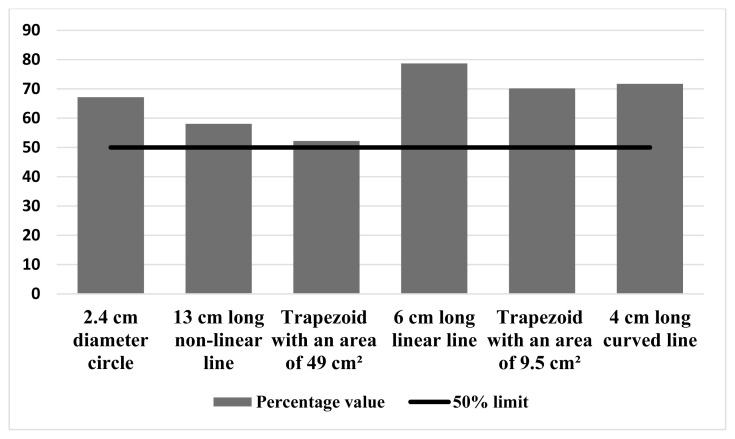
Percentage of values given to figures above their exact value.

It was observed that more than half of the participants gave values above the actual length or area value of each shape. The percentage of values given to each shape above its real value is shown in Figure 2.

The values given for the figures were categorised as correct and incorrect estimates with ±10% margin of error. The number and proportion of interns and physicians correctly estimating the length or area of the figures with ±10% margin of error are shown in Table 3.

There was a significant difference (x^2^ = 5.1, p = .024) between physicians and intern physicians and the correct estimation of the circle with a diameter of 2.4 cm with a ±10% margin of error, but no statistical significance was found between the correct estimation of the length or area of other shapes with a ±10% margin of error.

According to the results of Spearman correlation analysis between the age of the participants and the correct estimation of the length or area of the shapes with ±10% margin of error, a positive correlation was found with a circle with a diameter of 2.4 cm (r = .107, p = .017) and a negative correlation with a nonlinear line of 13 cm (r = −.110, p = .015), but no significant correlation was found between the length and area estimation of other shapes. According to spearman correlation analysis, no significant correlation was found between the professional duration of the physicians and the correct estimation of the length or area of the shapes with ±10% margin of error.

It was observed that the rate of estimating the length of the shapes correctly with ±10% margin of error was higher than the rate of estimating the area of the shapes correctly with ±10% margin of error. The proportion of those who correctly guessed the 13 cm long nonlinear line with ±10% margin of error was higher than the proportion of those who correctly guessed the other two line shapes with ±10% margin of error.

## 4. Discussion

Injuries resulting from traumatic situations; these are the cases that surgeons, emergency physicians and forensic medicine physicians frequently encounter in their daily practices. Defining, guiding and reporting the treatment includes features that differ for each case. Incorrect measurement of wound size may affect the treatment method to be applied. In addition, such evaluation/measurement errors in the judicial report to be prepared may cause loss of rights of the injured or injured person.

To prevent possible errors during the preparation of forensic reports in our country and to standardise forensic reports, a guideline titled “Evaluation of Injury Offences Defined in the Turkish Penal Code in terms of Forensic Medicine” has been prepared by the Presidency of the Forensic Medicine Institute, the Association of Forensic Medicine Specialists and the Forensic Medicine Association. In this guideline, limit length or area values have been determined to determine the aggravating reasons (whether/not to cause a life-threatening situation, whether/not to cause a minor injury that can be eliminated by a simple medical intervention, whether/not to cause a minor injury that can be eliminated by a simple medical intervention, permanent weakening or loss of the function of one of the senses or organs) in the relevant articles of the Turkish Penal Code for wounds such as incision, burn, hair loss, flap-style injury to the scalp, etc. Although there are various criticisms/suggestions to the cut-off points in the guide in studies on this subject, the importance of precise measurement values for determining the trauma score is undisputed [6, 13].

In our study, no participant correctly estimated all of the length and area measurements. As exact values, 122 (24.7%) participants correctly estimated a curvilinear line shape with a maximum length of 4 cm and 167 (33.8%) participants correctly estimated a nonlinear line shape with a maximum length of 13 cm with a ±10% margin of error. In a study conducted on schoolchildren, there are no studies on this subject in adults and professionals, but in a study conducted in children; it was reported that length estimation performed better than area estimation [14]. Similarly, in our study, it was observed that the rate of correct estimation of line shapes was higher than the rate of correct estimation of area shapes both as exact values and with ±10% margin of error.

Bourne et al. [11] showed that the majority of the participants made higher estimates than the actual value of the lesions in their study with lesion simulation on pig cadavers. Similarly, in our study, it was determined that more than half of the participants gave values above the actual length and area values of the shapes for all shapes (Figure 2). It is understood that people tend to give higher values when giving an estimate. It was found that correct estimation decreased as the complexity and length of the lesion or the shape simulating the lesion increased [2, 11]. Similarly, in our study, it was observed that the number of those who gave exact values decreased as the line length increased in line shapes.

The average of the values given by the interns to each shape was found to be higher than the average of the values given by the physicians to each shape and more distant from the true value. Siegel et al. reported that children in higher grades were more successful in measurement estimation than children in lower grades [15]. In our study, it was thought that the deviation of the estimates of intern physicians from the true value more than the estimates of physicians was related with the fact that their professional experience was less than that of physicians or with age.

While discussing the accuracy and usability of the existing techniques developed in wound measurement, Umbrello et al. [16] showed that more than 90% of physicians do not regularly use a measuring device to document the length of laceration [17].

Although the simple ruler method often overestimates the wound area and is an inappropriate tool for large wounds with irregular borders, it is still an important measurement tool because it is fast, easy to use and inexpensive. [18].

In recent years, since it is predicted that 3D measurement approaches will provide more accurate results than two-dimensional measurement methods in the evaluation of biological variables in wound healing, methods that can provide 3D measurement have also started to be developed. However, studies have shown that 3D measurement technologies do not yet have an important place due to high cost, complexity in system installation and lack of focus on feasibility in the studies [19].

Morris et al. [20], in which physicians were asked to estimate the lengths of wounds simulated by drawing on a volunteer person via digital photographs and face-to-face, the accuracy of length estimates was found to be quite low in both methods, although the accuracy of face-to-face estimates was higher. Although the estimation of shapes as exact values and estimation with ±10% margin of error showed some differences according to gender, age, being an intern physician or a physician and years of professional experience, it was observed that the estimated values were quite inaccurate in all conditions. In a study conducted by Turla et al. [9] it was reported that the presence or absence of external traumatic lesions was not written in approximately 30.5% of the forensic reports and in almost half (48.1%) of the cases in which external lesions were described (69.5%), the lesion descriptions were not detailed as required in a forensic report. These results show that although the lesion definition is made as it should be in a forensic report, if a measuring instrument is not used in the definition, the forensic report may be quite inaccurate.

## 5. Limitations and strengths of the study

It is predicted that the individual past experiences of physicians in estimation affect the accuracy of the current estimation rates. The limitation of our study is that we could not obtain data to objectively evaluate the past experiences of physicians in estimation.

One of our limitations was that participants were asked to estimate the length or area of perspective-limited two-dimensional shapes, rather than 3D, complexly shaped, jagged lesions such as trauma-related wounds. However, if we cannot accurately predict simple shapes, it can be assumed that we will be less successful in predicting more complex lacerations. Another limitation of our study is that approximately half of the participants were interns with little professional experience. Although there are differences in the forensic medicine procedures of the countries, the universality of our study was preserved since lesion description and definition is needed in every field of medicine. Although there are a limited number of studies on the effect of length and area estimation on billing, our study is valuable in terms of being the first study to examine its effect on forensic medicine and raising awareness on this issue.

## 6. Conclusion

In addition to treating the patient, every physician also has the responsibility to prepare a report in forensic cases. The forensic report prepared in order to prevent the victimisation of the parties and to protect their rights in a case that has been reflected to the judicial authorities must be complete, capable of answering all possible forensic questions, and that the information accurately reflects the truth. Since even a single lesion that is not defined correctly may lead to erroneous results, estimated data should not be used while preparing the forensic report and determining the lengths and areas of the lesions. It should not be forgotten that this erroneous situation will also cause problems in terms of health financing. More information should be given in in-service training and medical education on the use of measuring instruments when describing lesions in forensic reports, how to describe lesions correctly, and the forensic, medical and financial consequences of incorrect lesion descriptions. It would also be useful to develop tools and equipment that will enable physicians to make practical measurements, such as a portable ruler for easy use, a pen with a ruler feature, and a forensic file with a measurement unit on it.

## Figures and Tables

**Table 1:** Comparison of the mean values given by interns and physicians.

	Intern physician n: 216				Physician n: 278				Total n: 494			
	mean	min	max	sd*	mean	min	max	sd*	mean	min	max	sd*
2.4 cm diameter circle	3.19	0.6	14	1.64	3.05	1	12	1.63	3.11	0.6	14	1.63
4 cm long curved line	4.75	1.5	11	1.64	4.51	1	15	1.72	4.62	1	15	1.69
6 cm long linear line	7.34	3	24	2.37	7.13	3	20	2.47	7.22	3	24	2.43
13 cm long non-linear line	14.18	5	32	4.2	14.02	5	45	5.23	14.1	5	45	4.8
Trapezoid with an area of 9.5 cm^2^	16.39	1.5	60	9.52	14.7	1.5	90	11.12	15.44	1.5	90	10.48
Trapezoid with an area of 49 cm^2^	59.38	7	360	43.59	58.2	5	300	43.21	58.71	5	360	43.34

*: Standard deviation.

**Table 2:** Distribution of the predicted values according to the ratio of exact values and incorrect values.

	Exact values		Incorrect values	
	n	%	n	%
2.4 cm diameter circle	3	0.6	491	99.4
4 cm long curved line	122	24.7	372	75.3
6 cm long linear line	107	21.7	387	78.3
13 cm long non-linear line	41	8.3	453	91.7
Trapezoid with an area of 9.5 cm^2^	1	0.2	493	99.2
Trapezoid with an area of 49 cm^2^	14	2.8	480	97.2

**Table 3:** Correctly estimating the length or area of shapes with ±10% margin of error.

Correctly estimating the length or area of shapes with ±10% margin of error	Intern physician n:216	Physician n:278	Total n:494
	n (%)	n (%)	n (%)
2.4 cm diameter circle	20 (9.3)	45 (16.2)	65(13.2)
4 cm long curved line	56 (25.9)	70 (25.2)	126(25.5)
6 cm long linear line	45 (20.8)	79 (28.4)	124(25.1)
13 cm long nonlinear line	83 (38.4)	84 (30.2)	167(33.8)
Trapezoid with an area of 9.5 cm^2^	28 (13.0)	39 (14.0)	67(13.6)
Trapezoid with an area of 49 cm^2^	42 (19.4)	45 (16.2)	87(17.6)
